# Exploring effect of herbal monomers in treating gouty arthritis based on nuclear factor-kappa B signaling: A review

**DOI:** 10.1097/MD.0000000000037089

**Published:** 2023-02-02

**Authors:** Zhanghao Guo, Guisheng Ye, Chengjian Tang, Hui Xiong

**Affiliations:** a Hunan University of Chinese Medicine, Changsha, People’s Republic of China; b Department of Ophthalmology, The First Hospital of Hunan University of Chinese Medicine, Changsha, People’s Republic of China.

**Keywords:** gouty arthritis, nuclear factor-kappa B, traditional Chinese medicine monomer

## Abstract

Gouty arthritis (GA) is an inflammatory disease caused by disorders of the purine metabolism. Although increasing number of drugs have been used to treat GA with the deepening of relevant research, GA still cannot be cured by simple drug therapy. The nuclear factor-kappa B (NF-κB) signaling pathway plays a key role in the pathogenesis of GA. A considerable number of Chinese herbal medicines have emerged as new drugs for the treatment of GA. This article collected relevant research on traditional Chinese medicine monomers in the treatment of GA using NF-κB, GA, etc. as keywords; and conducted a systematic search of relevant published articles using the PubMed database. In this study, we analyzed the therapeutic effects of traditional Chinese medicine monomers on GA in the existing literature through in vivo and in vitro experiments using animal and cell models. Based on this review, we believe that traditional Chinese medicine monomers that can treat GA through the NF-κB signaling pathway are potential new drug development targets. This study provides research ideas for the development and application of new drugs for GA.

## 1. Introduction

Gouty arthritis is a disease in which blood uric acid increases due to abnormal uric acid metabolism and/or purine metabolism in the body, causing monosodium urate (MSU) and other substances to accumulate in joints throughout the body, mainly manifested by redness, swelling, heat, and pain in joints, etc.^[1–3]^ As the disease progresses, gouty arthritis (GA) can lead to bone erosion, joint destruction, and loss of limb function.^[4]^ A global survey found that the global age-standardized disability-adjusted life years caused by GA were 24.61/100,000 person-years for men and 7.84/100,000 person-years for women.^[5]^ Because direct death from GA is relatively rare, disability-adjusted life years can be regarded as years lived with disability due to disability caused by GA.

With the deepening of research on GA, the pathogenesis of GA has gradually become clearer. Related studies have found that the classic pathway in the nuclear factor-kappa B (NF-κB) signaling pathway plays a decisive role in the development of GA.^[6]^

The main therapeutic goal of GA is to control the inflammatory response and uric acid levels in the body. Currently, the main treatment drugs include colchicine, nonsteroidal anti-inflammatory drugs, febuxostat, and allopurinol.^[7]^ Although the efficacy of these drugs is acceptable, gastrointestinal reactions, liver and kidney damage, and bone marrow suppression caused by taking these drugs have brought great pain to patients.^[8–10]^ Therefore, the use of new therapeutic drugs to improve the symptoms of GA patients has become a hot topic in modern medicine.

The main characteristics of GA include an increase in uric acid and the subsequent deposition of MSU in the limb joints of the body.^[11]^ In a state of high uric acid, MSU crystals can cause joint tissues to secrete inflammatory substances such as interleukin-8 (IL-8), Cyclooxygenase-2 (COX-2), and Nitrogen Monoxide (NO). These substances can recruit neutrophils to inflamed tissues and cause an inflammatory cascade reaction, leading to redness, swelling, heat, and pain in joints.^[12]^ The NF-κB signaling pathway is activated during the inflammatory process, causing the release of downstream inflammatory factors such as, interleukin-1β (IL-1β) and interleukin-18, amplifying the inflammatory cascade reaction.^[13]^

Studies have shown that GA is closely associated with the inflammatory and immune responses.^[14]^ Regulating of the inflammatory response to GA through the NF-κB signaling pathway has become a new treatment strategy. Traditional Chinese medicine (TCM) has a long history of GA treatment and has remarkable efficacy. With the development of bioinformatics, TCM has gradually been praised for its advantages of multi-component, multi-target, multi-pathway, reliable efficacy, and fewer side effects. Based on this, we sorted out relevant information regarding the use of TCM monomers to treat GA through the NF-κB signaling pathway, with the aim of providing a theoretical basis for the treatment of GA with TCM (Table 1).

**Table 1:** Chinese medicine monomers treat gouty arthritis through NF-κB signaling pathway.

Chinese medicine monomers	Mechanism	In vivo	In vitro
neoisoastilbin	IL-1β↓,IL-6↓,TNF-α↓,IKKα↓, NF- κB↓,IκBα↓, NLRP3↓,caspase-1↓,ASC↓	C57BL/6 mice	NA
Berberine	TLR4↓,NF-κB↓,NLRP3↓,PYCARD↓,CASP1↓,IL-1β↓,IKKα↓,IκBα↓	NA	peripheral blood mononuclear cells (PBMCs)
Palmatine	IL-1β↓,IL-6↓,IL-18↓,TNF-α↓,SOD↑,GSH↑, MDA↓,*P*-65↓,IκBα↓,NLRP3↓,ASC↓,Caspase-1↓	KM mice	THP-1 macrophages
Cynarin	IL-1β↓,IL-6↓,iNOS↓,TNF-α↓,p-p65↓,p-IKKa/β↓, NLRP3↓	C57BL/6 mice	Bone marrow-derived macrophages (BMDMs)
Carvacrol	CRP↓,NLRP3↓,NF-κB↓,TNF-α↓	Sprague-Dawley rats	NA
Epicatechin	IL-1β↓,IL-6↓,TNF-α↓,IL-18↓,p-IKKα↓,p-IκBα↓, p-p65↓,NLRP3↓	C57BL/6 mice	THP-1 macrophages
Calycosin	p62↑,AIM2↓,caspase-1↓,ASC↓,IL-1β↓,Keap1↓, p65↓,IκBα↓	C57BL/6 mice	peripheral blood mononuclear cells (PBMCs) and THP-1 macrophages
Neoastilbin	IL-1β↓,IL-6↓,TNF-α↓,p-p65↓,IKKα↓,p65↓,IκBα↓, NLRP3↓,Caspase-1↓,ASC↓	C57BL/6 mice	THP-1 macrophages
Curcumin	IL-1β↓,IL-6↓,TNF-α↓,p65↓,iNOS↓, IκBα↑, COX-2↓,PGE2↓,ROS↓,NLRP3↓,MPO↓,p65↓, p50↓	CD-1 mice and C57BL/6 mice	THP-1 macrophages and RAW264.7 cells
Paeonol	IL-1β↓,caspase-1↓,NLRP3↓,p-IKK↓,p-IκBα↓,p-p65↓, TNF-α↓, IL-6↓, NF-κB↓	Sprague-Dawley rats	J774A.1 cells
Total glucosides of paeony	IL-1β↓,TNF-α↓,NLRP3↓,Caspase-1↓,TLR4↓, MyD88↓,p-p65↓,p65↓,p-IkBα↓,IkBα↓	NA	THP-1 macrophages
β-Caryophyllene	NLRP3↓,Caspase-1↓,ASC↓,TLR4↓,MyD88↓,p65↓, IL-1β↓	Sprague-Dawley rats	NA
Dioscin	IL-1β↓,IL-6↓,TNF-α↓,NLRP3↓,ASC↓,caspase-1↓, MyD88↓,p-IKKβ↓,p-p65↓,NF-κB↓,	C57BL/6 mice	CM-H094
Icariin	IL-1β↓,IL-6↓,TNF-α↓,PGE 2↓,NLRP3↓,Caspase-1↓,ASC↓,MyD88↓,p65↓,IkBα↓	SD rats	NA
Dioscorea collettii	TLR4↓,NF- κB↓,MyD88↓,IL-1β↓,TNF- α↓	Wistar rats	THP-1 macrophages
Cichoric Acid	p65↓,IκBα↓,IL-1β↓,TNF- α↓,COX-2↓,PGE2↓	NA	THP-1 macrophages
Luteolin	IL-1β↓,IL-6↓,TNF-α↓,TLR2↓,TLR4↓,MyD88↓, NF-κB↓	Wistar rats	NA
Ferulic acid	TNF-α↓,IL-1β↓,NLRP3↓,caspase-1↓,p65↓,SOD↓, CAT↓	Wistar albino rats	NA

AIM2 = Absent In Melanoma 2, ASC = apoptosis-associated speck-like protein containing CARD, caspase-1 = cysteinyl aspartate specific proteinase-1, CAT = catalase, CRP = C-reactive protein, GSH = glutathione, IL-6 = interleukin-18, iNOS = Inducible Nitric Oxide Synthase, Keap1 = Kelch-like ECH-associated protein 1, MDA = malondialdehyde, MPO = myeloperoxidase, MyD88 = myeloid differentiation factor 88, NLRP3 = yrin Domain Containing Protein 3, PYCARD = apoptosis-associated specklike protein containing a CARD, ROS = reactive oxygen species, SOD = superoxide dismutase, TLR4 = Toll-likereceptor4, TNF-α = tumor necrosis factor-α.

## 2. Methods

This article reviews relevant articles on traditional Chinese medicine monomers that treat GA through the NF-κB signaling pathway. Articles published in peer-reviewed scientific journals were included in the study. Articles were excluded if they were written in a language other than English or were not published in peer-reviewed scientific journals.

### 2.1. Search strategy

The time span of the literature search was from the establishment of the literature database to October 2023. Relevant articles were identified using the PubMed database. The search keywords included gout, gouty arthritis, Chinese medicine, Chinese herbal medicine, traditional Chinese medicine, NF-κB, and hyperuricemia. The retrieval strategy was constructed by combining subject words with free words. This review includes relevant research on GA and Chinese herbal medicine monomers and the NF-κB signaling pathway.

### 2.2. Literature inclusion and exclusion criteria

#### 2.2.1. Inclusion criteria.

Research type: In vivo and in vitro experiments; type of disease studied: Gouty arthritis; language: limited to English. Intervention measures: The therapeutic drugs in the treatment group were Chinese medicine monomers, and the control group was not limited.

#### 2.2.2. Exclusion criteria.

The intervention measures in the treatment group were not traditional Chinese medicine, the language was not English, the full text could not be obtained, repeated publications, data were obviously wrong and incomplete, meta-analysis, systematic review, etc.

### 2.3. Literature inclusion status

Ninety-three relevant articles were obtained from the initial search. After reading the titles and abstracts of the articles, 42 were eliminated. We downloaded the full texts of the remaining articles and continued reading them. Finally, 18 articles were selected for discussion and analysis.

### 2.4. NF-κB signaling pathway and gouty arthritis

**Figure 1.**
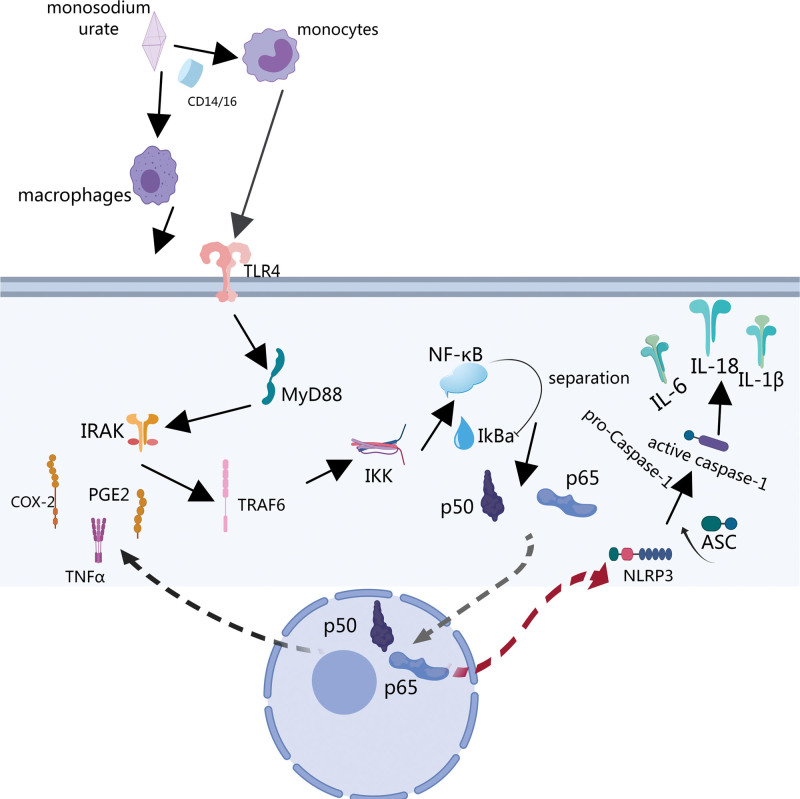
Schematic diagram of NF-κB signaling pathway related to gouty arthritis.

NF-κB mediates inflammation and energy failure, and regulates the production of inflammatory factors.^[15]^ Under normal conditions, NF-κB and its inhibitor IκBα bind to each other in the cytoplasm. When the body’s uric acid level increases, MSU acts as a danger signal and can bind to CD14/16 on the surface of monocytes/macrophages to stimulate Toll-like receptor 4 (TLR4).^[16,17]^ TLR4 interacts with the carboxyl terminus of myeloid differentiation factor (MyD88) to recruit interleukin-1 receptor-associated kinase. Interleukin-1 receptor-associated kinase is activated and acts on TNF receptor-associated factor 6. The inhibitor of kappa B kinase complex is activated, causing IκBα to be phosphorylated and rapidly degraded, separating it from NF-κB. NF-κB is then transferred to the nucleus to enhance the transcription of inflammation-related genes and plays a key role in regulating the release of inflammatory factors^[18]^ (Fig. 1). In summary, inhibition of the expression and activation of NF-κB can reduce the release of inflammatory mediators induced by MSU and alleviate the inflammatory response to GA.

## 3. Results

### 3.1. Flavonoids

#### 3.1.1. Neoisoastilbin and neoastilbin.

Neoisoastilbin (NIA) and neoastilbin are both derived from the Liliaceae plant, Smilax poria. Modern pharmacology has found that they have uric acid-lowering, anti-inflammatory, analgesic, and anti-tumor effects.^[19]^ Therefore, NIA can be used to treat GA, hypertension, tumors, and other diseases.^[20]^ In addition, NIA and neoastilbin were also found to reduce the secretion of IL-1β, IL-6, NO and the protein expression of NF-κB p-p65 in RAW264.7 cells induced by lipopolysaccharide.^[21]^ At the same time, Wang Y et al found that NIA and neoastilbin can alleviate joint swelling and inflammatory cell infiltration in AGA mice, and its mechanism of action may be achieved by simultaneously inhibiting the NF-κB/NLRP3 pathway and the expression of inflammatory factors.^[22,23]^ Therefore, the use of NIA and neoastilbin to regulate inflammation and oxidative stress responses should be the focus of future research on the treatment of GA.

#### 3.1.2. Calycosin.

Calycosin is one of the main components of astragalus flavonoids and has pharmacological activities such as scavenging oxygen free radicals, enhancing immunity, and antiviral activity.^[24–26]^ Tian et al^[27]^ demonstrated that calycosin can reverse MSU-accelerated recruitment of inflammatory cells and neutrophils in vivo and reduce lactate dehydrogenase content in vitro. Tian et al^[27]^ believes that calycosin can inhibit AIM2 inflammasome-mediated inflammation and pyroptosis through the NF-κB and p62-Keap1 pathways, and ultimately play a protective role in gouty arthritis. In general, it is indispensable that traditional Chinese medicine monomers can control the inflammatory response to GA through the NF-κB signaling pathway, but it is also inseparable from the role of pyroptosis.

#### 3.1.3. Icariin.

Icariin (ICA) is the main active ingredient in shorthorned epimedium herbs.^[28]^ It has pharmacological effects such as anti-inflammatory, anti-tumor, anti-oxidative stress and anti-tumor effects.^[29–32]^ In an experiment conducted by Cao,^[33]^ he found that ICA could reduce the swelling rate of ankle joints in GA rats, the expression of inflammatory factors in synovial tissue, and inhibit the nuclear translocation of NF-κB pathway-related proteins. In cell experiments, ICA also reduced chondrocyte activity, inflammatory factors, and glycosaminoglycan levels, and inhibited the nuclear translocation of NF-κB pathway-related proteins. This also indicates that ICA may be a new direction for the development of new GA drugs in the future.

#### 3.1.4. Luteolin.

Luteolin is a kind of phytochemical, which mainly exists in vegetables and fruits, and also exists in the form of glycosides in Chinese medicine.^[34]^ More and more studies have shown that luteolin can be used to treat gouty arthritis, diabetes, tumors, and cardiovascular and cerebrovascular diseases.^[35–39]^ Researchers found that luteolin can reduce joint synovial hyperplasia, cartilage, and bone erosion in GA rats.^[40]^ Luteolin reduces the expression of MyD88 by regulating TLR4 receptors, thereby inhibiting the expression of NF-κB-related proteins, reducing the release of related inflammatory factors, and alleviating the inflammatory cascade, thereby protecting joints.^[40]^

#### 3.1.5. (-)-Epicatechin.

(-)-Epicatechin (EC) is a catechin compound known for its rich and diverse biological and pharmacological activities^.[41]^ Wu et al^[42]^ showed that the expression of inflammatory factors in THP-1 cells induced by MSU and LPS increased, and joint swelling in MSU-induced GA mice was obvious. As a result, after EC intervention, the secretion of inflammatory cytokines was down-regulated by inhibiting the expression of NF-κB-related proteins.^[42]^ This suggests that EC may be a promising active ingredient in the prevention and treatment of gouty arthritis.

In summary, NIA, neoastilbin, calycosin, ICA, Luteolin, and EC are flavonoids. Neoastilbin and NIA have similar structures. They are both flavanols, which are mainly composed of 2 groups: a pyran ring and phenolic hydroxyl groups connected by molecules. Among them, the isopentenyl and ketone groups are the main components that ensure the pharmacological activities of NIA and neoastilbin, such as lowering uric acid, and anti-inflammatory and analgesic activities.^[43]^ Calycosin is composed of a flavonoid core and multiple hydroxyl and methoxy groups. The benzene ring and two isoprene units that constitute the flavonoid core also ensure multiple pharmacological activities of calycosin.^[44]^ ICA is an 8-prenyl flavonoid glycoside. The presence of an isoprenyl group on C-8 in its molecular structure is important to ensure its anti-inflammatory, antioxidant, and other effects.^[45,46]^ Luteolin is a natural pigment with a basic chemical structure that includes phenyl rings, flavonols with multiple double bonds, and an epoxy group. Its antioxidant activity and other pharmacological effects are widely believed to be affected by the 1,4-pyrone moiety and the 3-OH group.^[47]^ EC is a non-ester catechin with a basic flavanol-3-ol molecular structure. Its basic structure is 2-phenylbenzopyran, which is a polycyclic macromolecular structure with low symmetry. The polyhydroxyl structure present in each ring also ensures lipid-lowering, anti-inflammatory, and antioxidant properties of ECs. Pharmacological activity.^[48,49]^ It can be seen that flavonoids are interesting molecules synthetized by plants. They play an indispensable role in antioxidant and inflammatory responses.^[50,51]^ Since these flavonoids can be obtained from natural medicinal plants, this also opens up a new direction for the auxiliary treatment of certain diseases.^[52]^

### 3.2. Alkaloids

#### 3.2.1. Berberine.

Berberine (BBR) is found in Coptis chinensis, Phellodendron cypress and other plants. It is a natural isoquinoline alkaloid.^[53]^ Many studies have found that berberine has various pharmacological effects, such as inhibiting inflammation, antioxidant, anti-endothelial damage, and anti-tumor effects.^[54–56]^ It is well known that the initiation, amplification and resolution of acute inflammation induced by MSU and the accumulation of MSU leading to chronic inflammation and damage of joints are the most important core processes in the pathophysiology of gouty arthritis.^[57]^ Dang et al^[58]^ found in their study that MSU can promote the progression of GA inflammation by activating the NF-κB signaling pathway and NLRP3 inflammasome, while BBR can improve the inflammatory response, which may be related to the regulation of NF-κB signal transduction.

#### 3.2.2. Palmatine.

Palmatin (PAL) is similar to BBR and is an alkaloid belonging to the genus Coptis. It has various pharmacological properties, such as antibacterial and anti-inflammatory activities, liver and kidney protection, and uric acid-lowering effects.^[59]^ Researchers have found that PAL decreases the mRNA expression and levels of pro-inflammatory cytokines in a dose-dependent manner. Superoxide dismutase (SOD) and glutathione (GSH) levels were remarkably enhanced, while malondialdehyde levels were reduced.^[60]^ At the same time, it inhibits the NF-κB/NLRP3 signaling pathway by inhibiting the phosphorylation of *P*-65 and IκBα and blocking the expression of NLRP3, ASC, IL-1β, and Caspase-1.^[60]^ In summary, PAL prevents MSU-induced inflammation and oxidative stress by regulating the NF-κB signaling pathway, and is a potential candidate drug for the treatment of gouty arthritis.

It can be seen that BBR and PAL, as natural isoquinoline alkaloids, have similar effects and have played a good role in the treatment of GA. Berberine is a typical cyclic steroid compound, mainly composed of benzene rings, pyridine rings, methoxyphenyl groups, malonic acid groups, and other oxygen atoms. These complex groups and thedifferent bonding methods contribute to the diverse pharmacological activities of berberine.^[61]^ PAL is a nitrogen-containing heterocyclic compound whose main chemical structure includes a benzene ring, furan ring, alcohol group and amide group.^[62]^ Among these, the furan and benzene rings ensure that PAL can exert pharmacological activities, such as lowering uric acid, anti-inflammatory, and antibacterial activities.

### 3.3. Bicyclic sesquiterpenoids

#### 3.3.1. β-caryophyllene.

**Figure 2.**
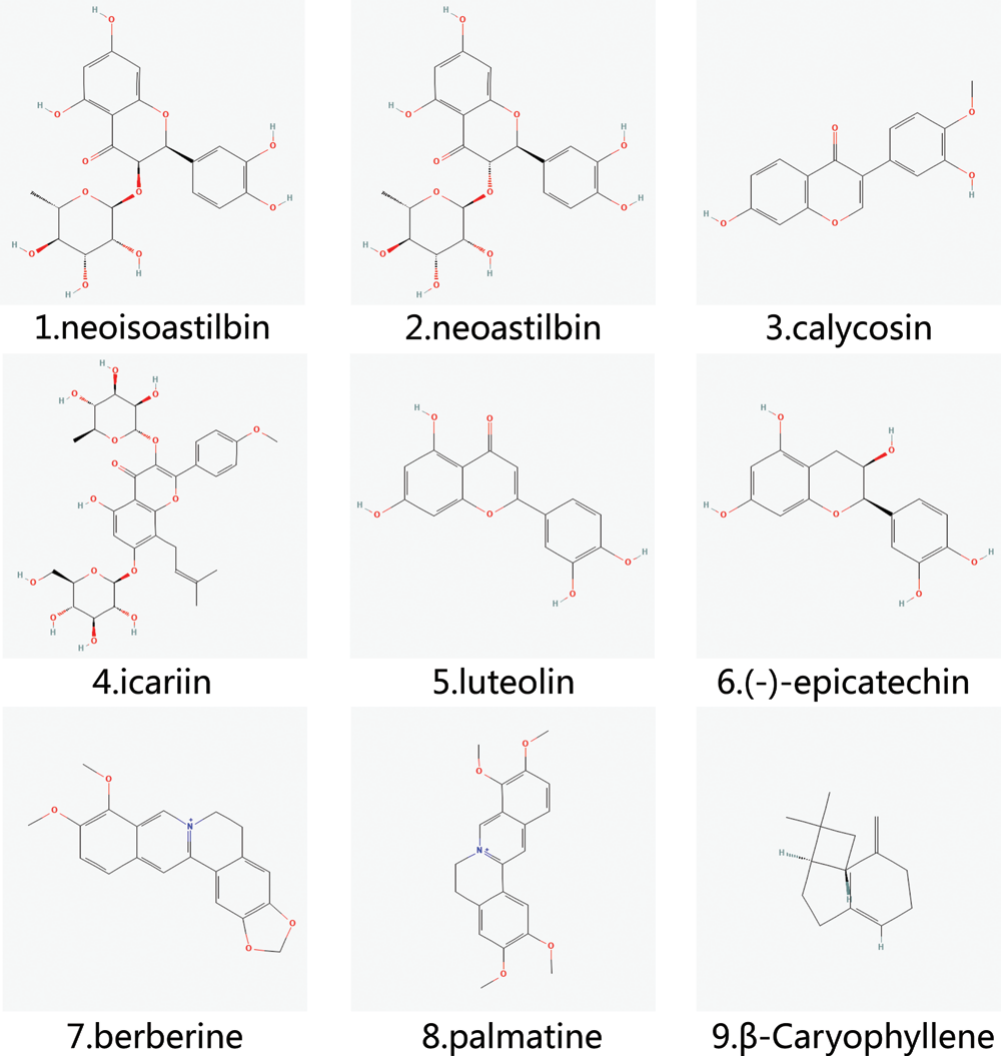
The chemical structures of flavonoids, alkaloids, and bicyclic sesquiterpenoids.

β-caryophyllene is a natural vegetable oil derived from cloves, cinnamon, and other plants. It also exhibits anti-inflammatory, antioxidant, and anxiolytic properties.^[63,64]^ Its chemical structure is mainly composed of five carbon rings, and its unique bicyclic sesquiterpene structure ensures its biological activity.^[65]^ Related studies have found that β-caryophyllene can significantly reduce inflammation and ankle joint function in MSU crystal-induced GA rats, while reducing serum cytokine levels. In addition, it can inhibit the expression of NLRP3, Caspase-1, ASC, TLR4, MyD88, p65, and IL-1β in synovial tissue, thereby reducing inflammation and protecting ankle joint function.^[66]^ This also provides new research avenues for the treatment of GA. The chemical structures of Flavonoids, Alkaloids, and Bicyclic sesquiterpenoids are shown in Figure 2.

### 3.4. Phenols

#### 3.4.1. Cynarin.

Cynarin (Cyn), also known as cynarin and cynaric acid, is a caffeoylquinic acid compound extracted from the traditional Chinese medicine cardoon. It can be used to treat inflammation, hypertension, tumors and other diseases.^[67,68]^ The researchers found that Cyn could alleviate joint swelling and M1 macrophage infiltration in GA mice.^[69]^ After treatment with Cyn, the expression of inflammatory factors and NF-κB-related proteins in MSU-induced GA mice decreased.^[69]^ In other words, Cyn can inhibit the inflammatory response induced by MSU through the NF-κB signaling pathway, improve inflammatory infiltration of the joint synovium, and protect the joints.

#### 3.4.2. Carvacrol.

Carvacrol is a monotetraphenol widely found in various natural plants^.[70]^ Recently, researchers have found that carvacrol exhibits antioxidant, anti-inflammatory, and anti-apoptotic activities.^[71,72]^ In fact, uric acid control is equally important during GA treatment. Riaz et al^[73]^ found that administration of carvacrol to GA rats for seven consecutive days showed significant anti-hyperuricemia effects in a dose-dependent manner. The expression of oxidative stress markers and NF-κB-related proteins was also reduced during treatment. This shows that carvacrol can reduce oxidative stress, reactions, and uric acid levels by mediating the NF-κB signaling pathway, thereby protecting against joint degeneration.

#### 3.4.3. Curcumin.

Curcumin (Cur) is a natural polyphenol extracted from turmeric rhizome. It is currently widely used in food additives, seasonings, etc.^[74]^ Cur is also widely used in diabetes, osteoporosis, cardiovascular diseases, and many cancers because of its anti-inflammatory, antioxidant, hypoglycemic, and anti-cancer properties.^[75]^ Relevant studies have found that Cur can reduce joint swelling, inflammatory cell infiltration, and MPO activity in MSU-induced AGA mice, which may be related to the inhibition of IκBα degradation, activation of the NF-κB signaling pathway, and expression of NF-κB downstream inflammatory genes such as IL- 1β, TNF-α, COX-2, and PGE2.^[76]^ However, problems such as rapid degradation, poor water solubility, and low bioavailability, limit the therapeutic effect of Cur.^[77]^ Zhang et al^[78]^ showed better drug stability and biocompatibility after loading free Cur with tetrahedral framework nucleic acids. At the same time, in subsequent in vivo and in vitro experiments, the newly synthesized Cur showed better anti-inflammatory effects than free Cur, which has great application prospects in the treatment of GA and similar inflammatory diseases.

#### 3.4.4. Paeonol.

Paeonol is a bioactive ingredient extracted from the peony bark. It has various pharmacological effects, such as inhibition of inflammatory response, antioxidation, and anti-tumor.^[79–81]^ IL-1β is a key cytokine in GA inflammation and is precisely regulated by the NF-κB and NLRP3 inflammasomes.^[82]^ Related experiments have shown that paeonol can inhibit the production of IL-1β in arthritic rats.^[83]^ Administration of paeonol to GA rats for seven consecutive days significantly reduced the expression of TNF-α, IL-1β, and IL-6 in the synovial tissue of rats. In addition, the levels of p65 in rat cell nuclei and NF-κB DNA-binding activity in synovial tissue were also reduced.^[84]^

#### 3.4.5. Cichoric Acid.

Cichoric Acid (CA) is a caffeic Acid derivative isolated from Echinacea purpurea (Linn.) Moench.^[85]^ Studies have proven that CA has various pharmacological activities such as anti-inflammatory, antioxidant, antibacterial, and immune enhancement.^[86,87]^ Finally, the expression of NF-κB-related proteins and their downstream inflammatory factors significantly decreased after CA treatment.^[88]^ This shows that CA is also a new therapeutic strategy to treat GA by regulating the NF-κB signaling pathway.

#### 3.4.6. Ferulic acid.

Ferulic acid (FA) is a phenolic acid extracted from traditional Chinese medicines, such as Asafoetida and Angelica. It has antioxidant and anti-free radical activities and is a precursor of many substances.^[89]^ In this study, Doss et al^[90]^ found that FA reduced the expression of elastase, lysosomal enzymes, nitric oxide, lipid peroxidation, pro-inflammatory cytokines, and NF-κB p65 in rats induced by MSU, while the expression of SOD and CAT Increased. This indicates that FA has potential anti-inflammatory effects in GA rats.

Phenolic compounds, such as Cyn, carvacrol, Cur, paeonol, CA, and FA, are effective in treating GA. Cyn is a 1,3-dicaffeoylquinic acid, composed of 2 benzene rings and 2 double bonds. The phenolic hydroxyl group on the benzene ring also ensured the activity of Cyn.^[91]^ Carvacrol is mainly composed of a benzene ring, epoxy ring, and methyl and hydroxyl groups on the ring. The presence of benzene and epoxy rings also ensures that carvacrol exerts antioxidant, anti-inflammatory, and other effects.^[92]^ The basic chemical structure of Cur is composed of two unsaturated ketone groups connected to a pyruvate group at the same center.^[93]^Such two benzene rings and an octadienone ring also ensure the basic pharmacological activity of Cur.^[94]^ Paeonol is composed of a benzene ring, hydroxyl group, carboxyl group, and pyran ring, in which a hydrogen atom on the benzene ring is replaced by a hydroxyl group, and an oxygen atom on the pyran ring forms an ester bond with the carboxyl group. CA is a typical hemiacetal compound. Its structure contains a -COOH group, which is the basic structure of CA. The hydroxyl groups in CA ensure that CA exerts its biological activity.^[95]^ FA is a cinnamic acid derivative, mainly composed of phenylpropionic acid phenolic hydroxyl, methoxy, and acrylic acid groups, among which the phenylpropionic acid phenolic hydroxyl group ensures the basic pharmacological activity of FA.^[96]^ From this, we can see that most of these phenolic compounds contain phenolic hydroxyl groups, which also makes them have good antioxidant and anti-inflammatory activities.

### 3.5. Glycosides

#### 3.5.1. Dioscin.

**Figure 3.**
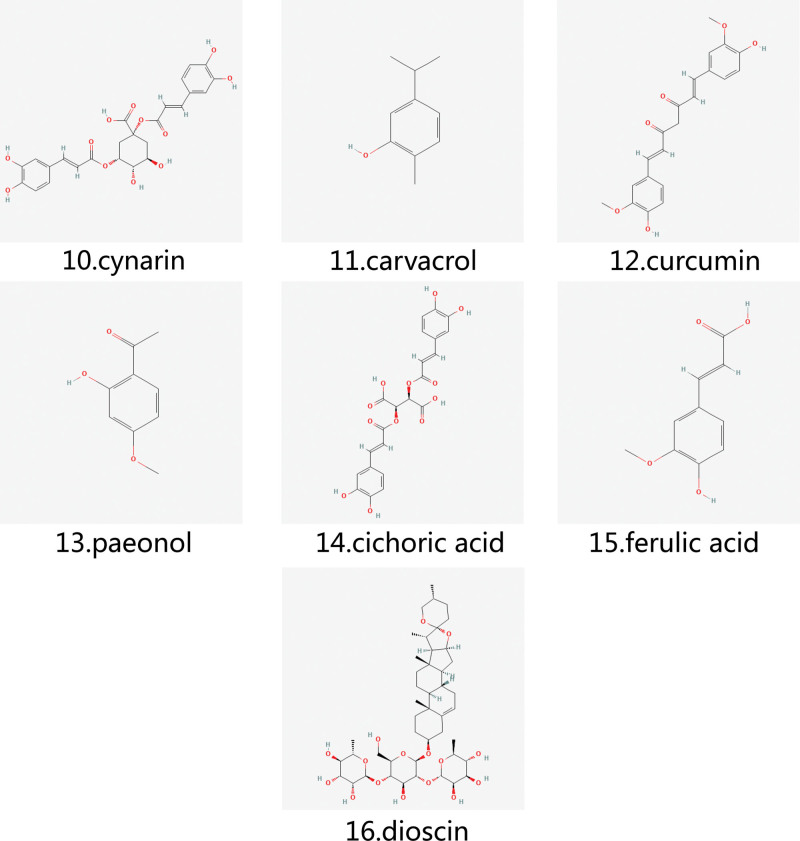
The chemical structures of phenols and glycosides.

Dioscin is a natural steroidal saponin that has pharmacological activities such as anti-inflammatory, antibacterial, hypoglycemic, anti-tumor, and blood lipid regulation.^[97]^ The main chemical structure of dioscin includes 4 ring structures, including one 6-membered ring, two 5-membered rings, and one 4-membered ring, which also ensures that dioscin can exert anti-inflammatory, antibacterial, anti-tumor, and other pharmacological activities. Han J found in vitro and in vivo experiments that dioscin can reduce the severity of GA, while reducing uric acid and creatinine levels, which may be related to the inhibition of inflammatory cytokine production and activation of the NF-κB signaling pathway.^[98]^ The chemical structural formulae of phenols and glycosides are shown in Figure 3.

## 4. Conclusion

From this, we can also see that NF-κB-related signaling targets have been investigated in many studies as potential ways to treat GA. Compared with the current main treatment drugs for GA, Chinese medicine monomers are gradually being favored by clinicians and patients because of their extremely mild side effects and good efficacy.^[99,100]^

Overall, NF-κB and its related targets have become a research hotspot in GA. Canonical NF-κB is activated by a variety of stimuli, has rapid transcription but short-lived activity, and can regulate the expression of various pro-inflammatory genes.^[101]^ As a key mediator of the inflammatory response, NF-κB has been regarded as an important therapeutic target for the treatment of inflammatory diseases.^[15]^ As an initiating signal, NF-κB combines with the promoter of the NLRP3 inflammasome after activation to promote the activation of Caspase-1, thereby regulating the production and secretion of TNF-α, IL-1β, IL-8 and other factors.^[102]^ Therefore, regulating the conduction of the NF-κB signaling pathway is helpful for the treatment of GA.

As a cultural product of the theory and practice of TCM for thousands of years, TCM has made significant contributions to the world’s medical and health undertakings. Many biologically active compounds form the basis of TCM treatment. The rational use of active ingredients in TCM to develop new drugs is a reasonable and rapid way to develop new drugs.^[103,104]^

In this era of data, this study lists relevant traditional Chinese medicine monomers for the treatment of GA through literature management. Simultaneously, the prospects and key targets of GA drug development were prospected. Of course, it should be acknowledged that this study has certain limitations: the inclusion of literature may not be comprehensive enough. This study only searched the PubMed database, and the language was limited to English. Other databases and non-English literature were not reviewed. All included studies were animal or cell experiments and did not involve clinical research. Further confirmation of these findings requires additional high-quality, multicenter, large-scale randomized controlled trials.

In summary, the use of Chinese medicine monomers to alleviate the inflammatory response to GA through the NF-κB signaling pathway has become a popular treatment option. We hope that this study will provide a theoretical basis for the treatment and drug development of GA.

## Acknowledgments

We would like to thank Dr Qi Hu for improving the language of this manuscript.

## Author contributions

**Conceptualization:** Zhanghao Guo, Guisheng Ye, Chengjian Tang.

**Methodology:** Guisheng Ye, Chengjian Tang.

**Resources:** Hui Xiong.

**Writing – original draft:** Zhanghao Guo.

**Writing – review & editing:** Zhanghao Guo.
